# Retinal venous pressure is decreased after anti-VEGF therapy in patients with retinal vein occlusion–related macular edema

**DOI:** 10.1007/s00417-020-05068-x

**Published:** 2021-01-15

**Authors:** Teruyo Kida, Josef Flammer, Katarzyna Konieczka, Tsunehiko Ikeda

**Affiliations:** 1 Department of Ophthalmology, Osaka Medical College, 2-7 Daigaku-machi, Takatsuki, Osaka 569-8686 Japan; 2 Department of Ophthalmology, University of Basel, Basel, Switzerland

**Keywords:** Anti-VEGF, Branch retinal vein occlusion (BRVO), Central retinal vein occlusion (CRVO), Macular edema, Ophthalmodynamometer, Retinal venous pressure (RVP)

## Abstract

**Purpose:**

The pathomechanism leading to retinal vein occlusion (RVO) is unclear. Mechanical compression, thrombosis, and functional contractions of veins are discussed as the reasons for the increased resistance of venous outflow. We evaluated changes in the retinal venous pressure (RVP) following intravitreal injection of anti-vascular endothelial growth factor (VEGF) agent to determine the effect on RVO-related macular edema.

**Methods:**

Twenty-six patients with RVO-related macular edema (16 branch RVOs [BRVOs] and 10 central RVOs [CRVOs], age 72.5 ± 8.8 years) who visited our hospital were included in this prospective study. Visual acuity (VA), intraocular pressure (IOP), central retinal thickness (CRT) determined by macular optical coherence tomography, and RVP measured using an ophthalmodynamometer were obtained before intravitreal injection of ranibizumab (IVR) and 1 month later.

**Results:**

Comparison of the BRVOs and CRVOs showed that VA was significantly improved by a single injection in BRVOs (*P* < 0.0001; *P* = 0.1087 for CRVOs), but CRT and RVP were significantly decreased without significant difference in IOP after the treatment in both groups (*P* < 0.0001).

**Conclusion:**

The anti-VEGF treatment resulted in a significant decrease in the RVP, but the RVP remained significantly higher than the IOP. An increased RVP plays a decisive role in the formation of macula edema, and reducing it is desirable.

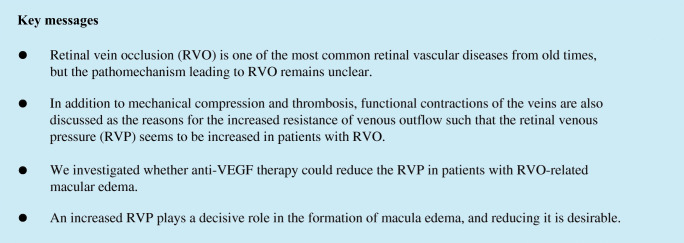



## Introduction

Branch retinal vein occlusion (BRVO) was first reported by Leber in 1877 [1], and central retinal vein occlusion (CRVO) by Michel in 1878 [2]. While BRVO commonly occurs in relation to arteriovenous crossing in the unilateral eye [3], CRVO is an obstruction of the central retinal vein and is believed to occur in the retrolaminar region of the optic nerve head [4]. Retinal vein occlusion (RVO) is one of the most common retinal vascular diseases from old times, but the pathomechanism leading to RVO remains unclear. The occlusions seem to be induced by an interaction between local and systemic factors [1, 5–8]. In addition to mechanical compression, the vein locally constricts by itself [9–11]. We can observe vascular changes, such as focal venous obstruction or venous narrowing. In addition to mechanical compression and thrombosis, functional contractions of the veins are also discussed as the reasons for the increased resistance of venous outflow such that the retinal venous pressure (RVP) seems to be increased in patients with BRVO and CRVO. Jonas reported that the RVP was significantly higher in eyes with CRVO and BRVO than in normal eyes [12], and Mozaffarieh et al. observed that the RVP is elevated not only in the eye with CRVO but, to some extent, also in the contralateral apparently healthy eye [13].

Macular edema is the major vision-threatening complication associated with RVO, and it often causes vision decline in patients with this disease. An impaired blood-retinal barrier and increased RVP are both involved in the formation of macular edema. The congestion of the vein can lead to local hypoxia, which, in turn, leads to an increase in hypoxia inducible factor-1-alpha (HIF-1) that also increases the expression of the vascular endothelial growth factor (VEGF) and endothelin, both contributing to macula edema [14, 15]. Anti-VEGF therapy leads to the rapid reduction of macular edema and an improvement in visual function. However, some patients with RVO require the repeated anti-VEGF drug injections. In some recurrent patients, therefore, there might be a possibility that the RVP is not reduced enough even after a single anti-VEGF drug injection. In addition, the measurement of RVP in patients with RVO-related macular edema might be helpful in predicting whether the patient needs more injections.

In this prospective study, we investigated whether anti-VEGF therapy could affect the RVP in patients with RVO-related macular edema. Under clinical conditions, we measured the RVP using an ophthalmodynamometer (Imedos, Jena, Germany) before and after injecting intravitreal ranibizumab (IVR). We measure RVP because we think it contributes to the clinical picture of RVO and because it may become a good follow-up parameter in the future.

## Methods

Twenty-six RVO patients with untreated macular edema (16 BRVOs and 10 CRVOs) were included in this study. Study approval was obtained from the Institutional Review Board at Osaka Medical College (Registration No. 2038-1) and the research adhered to the tenets of the Declaration of Helsinki.

The inclusion criteria of this study were as follows: (1) symptomatic BRVO or CRVO with macular edema; (2) a foveal thickness greater than 250 μm, as measured by optical coherence tomography (OCT) at the initial visit; and (3) macular edema secondary to RVO, which had never been previously treated. The diagnosis of RVO was based on the findings from fundus examinations and fluorescein angiographies. Eyes with any co-existing ocular diseases, such as age-related macular degeneration, diabetic retinopathy, hypertension retinopathy, or uveitis, were excluded.

At the initial visit, medical history, including disease durations, was obtained from each patient through a medical interview. At the initial examination, each patient underwent comprehensive ophthalmic examinations, which included the measurement of best-corrected visual acuity (VA) using a Landolt chart, measurement of intraocular pressure (IOP), and fundus biomicroscopy with a non-contact lens. Digital fundus photography, fluorescein angiography, and OCT examination were also performed after pupil dilation. The RVO present in each case was classified as ischemic if the fluorescein angiography revealed more than 10 disc areas of retinal non-perfusion for CRVO and 5 disc areas for BRVO.

All patients were treated by injection of IVR after their informed consent was obtained. At each visit before IVR and one month after IVR, each patient underwent a comprehensive ophthalmologic examination, including measurement of best-corrected VA, IOP, RVP, and OCT examination.

All values are presented as the mean ± standard deviation. For statistical analysis, VA measured with a Landolt chart was converted to the logarithm of the minimum angle of resolution (logMAR). Central retinal thickness (CRT) measured with OCT was determined as the average retinal thickness in a 1-mm-diameter circular region at the fovea. Student’s *t* tests were used to evaluate changes in the best-corrected VA (logMAR), CRT, and IOP.

## Results

In this study, we report on 26 eyes from 26 different patients with RVO-related macular edema that received treatment with IVR. Table 1 shows the demographic distribution in the present study. Before the treatment, all eyes showed retinal hemorrhage and macular edema associated with RVO. Ischemic RVO was suspected in three patients at the first visit. The total mean age was 72.5 ± 8.8, ranging from 50 to 87 years, with 72.4 ± 7.5 years for the BRVOs and 72.5 ± 11.0 years for the CRVOs. Three patients with normal tension glaucoma (NTG) (two BRVOs and one CRVO) and one primary open-angle glaucoma (POAG) patient with CRVO were included.

**Table 1:** Demographic distribution and changes of VA, IOP, and RVP in eyes with RVO

	Total (*n* = 26)	BRVO (*n* = 16)	CRVO (*n* = 10)
Age (years)	72.5 ± 8.8	72.4 ± 7.5	72.5 ± 11.0
Females/males	19/7	14/2	5/5
Pre VA	0.62 ± 0.39	0.58 ± 0.33	0.70 ± 0.48
Post VA	0.38 ± 0.31	0.32 ± 0.28	0.46 ± 0.36
Pre IOP (mmHg)	12.4 ± 2.3	12.3 ± 2.1	12.6 ± 2.6
Post IOP (mmHg)	12.6 ± 2.5	12.0 ± 1.9	13.6 ± 3.2
Pre RVP (mmHg)	40.0 ± 5.9	38.6 ± 7.2	42.2 ± 1.6
Post RVP (mmHg)	29.8 ± 9.0	27.4 ± 10.2	33.8 ± 5.1

*VA* visual acuity, *IOP* intraocular pressure, *RVP* retinal venous pressure, *pre* before the treatment, *post* after the treatment

**Fig. 1:**
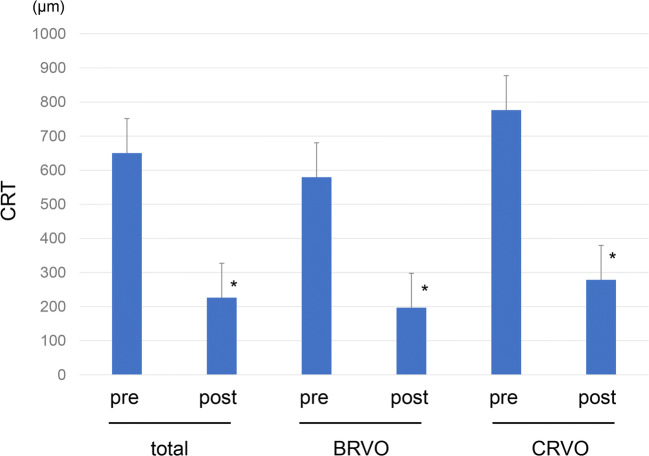
Changes in central retinal thickness (CRT) before and after treatment. The mean CRT was significantly decreased after treatment (*P < 0.0001, paired *t* test)

**Fig. 2:**
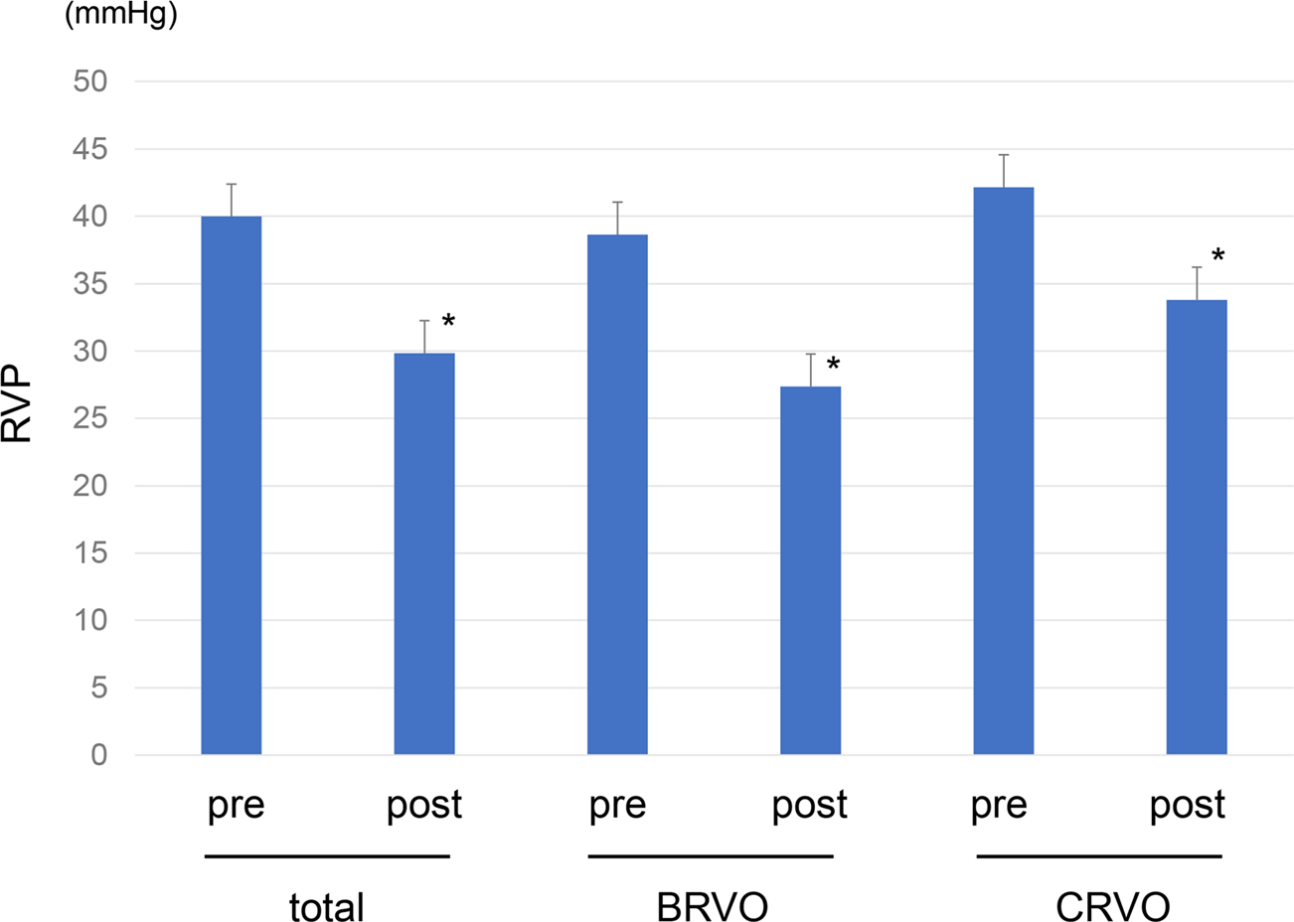
Changes in retinal venous pressure (RVP) before and after treatment. The mean RVP was significantly decreased after treatment (*P < 0.0001, paired *t* test)

Figure 1 shows the changes of CRT before and after the treatment. The CRT was significantly decreased in every group of the total RVOs, BRVOs, and CRVOs. The mean CRT was significantly decreased from 650.2 ± 237.4 μm before the treatment to 226.1 ± 72.4 μm 1 month after the initial injection (*P* < 0.0001, Fig. 1). The mean VA was significantly improved from 0.62 ± 0.39 before the treatment to 0.38 ± 0.31 1 month after the treatment (*P* = 0.0004, Table 1). The mean RVP level was significantly decreased after IVR from 40.0 ± 5.9 mmHg to 29.8 ± 9.0 mmHg (*P* < 0.0001, Table 1 and Fig. 2). There was no significant difference in IOP before and 1 month after the treatment (*P* = 0.7698, Table 1). Comparison of the BRVO and CRVO groups separately showed that VA was significantly improved from 0.58 ± 0.33 to 0.32 ± 0.28 in the BRVO group. In the CRVO group, VA was changed from 0.70 ± 0.48 before the treatment to 0.46 ± 0.36 1 month after the IVR (*P* = 0.1087). The mean CRT was 579.3 ± 166.5 μm before the treatment and 196.7 ± 25.3 μm 1 month after IVR in the BRVO group, whereas it was 776.2 ± 298.6 μm before the treatment and 278.3 ± 98.5 μm 1 month after IVR in the CRVO group. The mean RVP was 38.6 ± 7.2 mmHg before the treatment and 27.4 ± 10.2 mmHg after the treatment in the BRVO group, whereas it was 42.2 ± 1.6 mmHg before the treatment and 33.8 ± 5.1 mmHg after the treatment in the CRVO group. The CRT and RVP were significantly decreased in both the BRVO and CRVO groups (*P* < 0.0001, Figs. 1 and 2). There was no significant difference in IOP before and 1 month after the treatment in both the BRVO and CRVO groups (Table 1). In addition, the RVP in eyes with RVO was higher than the IOP both before and after the treatment.

## Discussion

In this study, a local anti-VEGF treatment for RVO-related macular edema was applied to the eyes of 26 patients. One month later, the CRT and RVP were significantly reduced in both the BRVO and CRVO groups, and VA was significantly improved in BRVO patients. We found that the RVP in eyes with RVO was significantly decreased after IVR. To the best of our knowledge, we are the first to show that an anti-VEGF therapy for RVO-related macular edema decreases RVP.

RVP is reported as a useful parameter in the management of ophthalmic and systemic disorders, such as glaucoma, increased intracranial pressure, RVO, and diabetic retinopathy [16–22]. In general, the RVP in normal eyes almost equals the IOP [23, 24]; however, it is becoming increasingly clear that RVP is often elevated in various diseases, and these are interestingly diseases in which RVOs are more common. In the present study, we showed that the RVP in eyes with RVO was significantly decreased by a single injection of anti-VEGF drug, but the RVP was still higher than the IOP. We need a long-term follow-up of RVP changes with the repeated injections.

It is known that a reduction in VEGF normally also leads to a reduction in macular edema with RVO. In the present study, we found that RVP, which is one of the local factors causing RVO, was decreased after anti-VEGF therapy in patients with RVO-related macular edema. As we already know, enhancement of RVP is secondary to RVO, the observation that in patients with RVO the RVP is also elevated in the contralateral eye [13] suggests that this RVP increase cannot only be secondary. It is therefore conceivable that, at least in certain cases, a high RVP precedes RVO and, by contributing to the local hypoxia, contributes to the development of RVO or may even cause it. In relation to systemic factors, lower doses of calcium channel blockers for systemic hypertension than the doses commonly used were found to reduce RVP in patients with retinal vein occlusion [25]. This indicates that systemic hypertension is a well-known risk factor for RVO, and the treatment of systemic hypertension is important for the improvement of RVO-associated macular edema [8]. Another report showed that the degree of RVP elevation was correlated with capillary nonperfusion in patients with severe CRVO [26]. McAllister et al. have also shown that patients with high RVP have a much worse prognosis (e.g., they develop rubeosis more often). Taken together, measuring the RVP in patients with RVO-related macular edema could be a prognostic factor for the recurrence of macular edema.

The pathomechanism of RVO remains unclear. It is known that endothelin-1 (ET-1) plasma is increased in patients with RVO. ET-1 induces constriction of retinal veins, both in vitro and in vivo, and plays a role in the regulation of RVP [7, 9, 27]. Interestingly, there are interactions between VEGF and ET-1. It has been reported that VEGF stimulates the production of ET-1 [28]. High ET-1 contributes to hypoxia, which, in turn, leads to an increased HIF-1 that increases the coexpression of ET-1 and VEGF [29]. In our previous study, we showed that anti-VEGF drug treatment reduced ET-1 levels in patients with BRVO [30]. Therefore, it is understandable that anti-VEGF therapy in patients with RVO can suppress VEGF and ET-1 levels and then lower the RVP. We recruited patients with RVO-related macular edema in the present study, and their macular edema (measured CRT, Fig. 1) was significantly resolved by anti-VEGF drug. As the RVP increases, the transmural pressure in the capillaries also increases. This increased transmural pressure might lead to macular edema in patients with RVO. If so, lowering the RVP in patients with RVO can also have an important role for macular edema.

This prospective study has some limitations. Its sample size was small, and its follow-up duration was short. Further investigations involving a long-term follow-up with repeated injections of anti-VEGF drug are needed. We also did not measure the VEGF level in intraocular fluid. It would be interesting to see whether the RVP increases again further down the treatment regimen despite ongoing anti-VEGF injections, or if ongoing anti-VEGF causes further lowering of the RVP. In addition, the lowering of RVP that is observed in this study after intravitreal anti-VEGF may be a natural course in eye with retinal vein occlusions, and not a direct effect of anti-VEGF treatment. It is known that the RVP naturally decreases with time after retinal vein occlusions without any intervention. To prove our hypothesis, we would need to prospectively compare the RVP in 2 groups of eyes with retinal vein occlusions, one receiving anti-VEGF injections and the other not receiving anti-VEGF injections. This, however, can be an ethical issue to withhold treatment from patients with macular edema. In this prospective study, we indicated that high RVP is possibly involved in the pathogenesis of RVO, and eyes with RVO after IVR showed decreased RVP levels though suppression of intraocular VEGF by anti-VEGF therapy. Moreover, three patients with NTG and one POAG patient with CRVO were included in the present study. We should have measured the RVP also in unaffected eyes so that the RVP in eyes treated with IVR became lower; however, the RVO-unaffected eyes with glaucoma might have remained to have high RVP in above glaucoma patients, as previously reported [20, 24].

In conclusion, we showed that a local anti-VEGF therapy for macular edema in patients with RVO led to a significant decrease in the RVP. An RVP level that is sufficiently suppressed by repeated anti-VEGF therapies or other treatments, such as the improvement of systemic hypertension, is thought to be important for patients with RVO-related macular edema. Measuring the RVP in eyes with RVO-related macular edema might have the potential to predict the requirements of anti-VEGF therapy.
